# Cost-effectiveness of an urinary biomarker panel in combination with MRI for prostate cancer diagnosis

**DOI:** 10.1007/s00345-023-04389-w

**Published:** 2023-05-03

**Authors:** Tim M. Govers, Matthew J Resnick, Ardeshir R. Rastinehad, Laura Caba, Jack Groskopf, Wim van Criekinge

**Affiliations:** 1 Department of Medical Imaging, Radboud University Medical Center Nijmegen, Geert Grooteplein-Zuid 10, 6500 HB Nijmegen, The Netherlands; 2 Department of Urology, Vanderbilt University Medical Center, Nashville, TN USA; 3 Embold Health, Nashville, TN USA; 4 The Smith Institute for Urology at Lenox Hill, Northwell Health, New York, NY USA; 5 MDxHealth Inc., Irvine, CA USA; 6 Department of Bioinformatics, Ghent University, Ghent, Belgium

**Keywords:** Cost-effectiveness, Prostate cancer, Biomarkers, Biopsy, Magnetic resonance imaging

## Abstract

**Purpose:**

The health impact and cost-effectiveness of the biomarker test SelectMDx were evaluated when used in combination with MRI, in two US populations: biopsy naïve men and men with a previous negative biopsy.

**Methods:**

Using a decision model, the current MRI strategy was compared with two SelectMDx strategies: SelectMDx used before MRI to select men for MRI and SelectMDx used after a negative MRI to select men for biopsy. Parameters were informed by the literature most relevant for both populations. Differences in quality-adjusted life years (QALYs) and costs between the current strategy and the SelectMDx strategies were calculated using two different assumptions regarding PCa-specific mortality (SPCG-4 and PIVOT).

**Results:**

In biopsy naïve men, the use of SelectMDx before MRI results in a gain of 0.004 QALY per patient under the SPCG-4 scenario, and a gain of 0.030 QALY under the PIVOT scenario. The cost savings are $1650 per patient. When used after MRI, SelectMDx results in a QALY gain per patient of 0.004 (SPCG-4), and 0.006 (PIVOT) with $262 in cost savings.

In the previous negative population, SelectMDx before MRI results in a QALY gain of 0.006 (SPCG-4) and 0.022 (PIVOT), with $1281 in cost savings per patient. SelectMDx after MRI results in a QALY gain of 0.003 (SPCG-4) and 0.004 (PIVOT) with $193 in cost savings.

**Conclusion:**

Application of SelectMDx results in better health outcomes and cost savings. The value of SelectMDx was highest when used before MRI to select patients for MRI and subsequent biopsy.

**Supplementary Information:**

The online version contains supplementary material available at 10.1007/s00345-023-04389-w.

## Introduction

Opportunistic prostate-specific antigen (PSA) testing is widely used to detect PCa at an early stage. Typically, men with elevated PSA are offered a systematic transrectal ultrasonography-guided biopsy of the prostate. The principal challenge of opportunistic PSA screening is its low specificity, resulting in unnecessary prostate biopsies, which confer risks of infection, hematuria and urinary retention [1, 2]. Furthermore, opportunistic PSA testing frequently results in the detection of PCa that likely would not cause any clinical consequence if left untreated, while treatment is costly and confers significant risks of both incontinence and impotence [3, 4].

Increasing evidence supports the use of magnetic resonance imaging (MRI) when used as a localization tool to guide MRI-targeted techniques since it increases the detection of aggressive (clinically significant) PCa [5–7]. The use of MRI is supported by most guidelines for patients with a previous negative biopsy and in some guidelines for biopsy-naïve patients [8–11].

While the use of MRI improves detection of clinically significant PCa, there remains a significant risk of PCa overdiagnosis and overtreatment [12]. Our previous study found that the application of a 2-gene biomarker test that targets *HOXC6* (cell proliferation gene) and *DLX1* (progression gene) improves health outcomes and lowers the costs associated with PCa when used to select patients for systematic biopsy [13]. This SelectMDx test can also be combined with MRI to optimize the population of men for whom biopsy is most indicated, thereby reducing overdiagnosis and overtreatment, while detecting the majority of clinically significant PCa. To this end, we evaluated health outcomes and cost-effectiveness of SelectMDx in combination with MRI strategies.

## Materials and methods

### Target populations

For this assessment two target populations were defined:

#### Biopsy naïve

US men with an initial clinical suspicion of PCa based on an elevated PSA and/or abnormal digital rectal exam (DRE). In a previous study, it was estimated that 311,879 men undergo initial prostate biopsy for elevated PSA and/or abnormal DRE, each year [13].

#### Previous negative biopsy

US men with continued suspicion of PCa after a previous negative systematic biopsy. It was estimated that yearly 26,853 men receive a repeat biopsy. This was based on 41% negative first biopsies with 21% of men with a first negative who receive repeat biopsy [12, 14].

Both population estimates are probably conservative as the total number of biopsies (including monitoring) was estimated to be over 1 million each year [15].

### Strategies

Within the two target populations, the current MRI strategy was compared with two strategies in which SelectMDx was used. Appendix A gives an overview of the current MRI strategies and the two SelectMDx strategies:

#### Current MRI strategy

For patients within both target populations (biopsy naïve and prior negative biopsy) MRI is performed. In men with a positive MRI, a systematic biopsy is then conducted, targeting suspicious lesions with additional cores (targeted biopsy). Positive MRI is defined as PI-RADS 3–5. For this assessment, we included MRI-TRUS fusion as the method for performing targeted biopsies, as this seems the most used method of targeted biopsy. Patients with a negative MRI (PI-RADS 1–2) underwent systematic biopsy only.

#### SelectMDx strategy 1

SelectMDx before MRI: Patients in both target populations undergo a SelectMDx test up front to select patients for MRI and subsequent biopsy. This means patients with a positive SelectMDx will then undergo MRI with the same consequence as described in the current MRI strategy. Patients with a negative SelectMDx do not move forward with either MRI or biopsy.

#### SelectMDx strategy 2

SelectMDx after negative MRI: Patient in both target populations first undergo MRI. Patients with a positive MRI (PI-RADS 3–5) will undergo biopsy as described in the current MRI strategy. Patients with a negative MRI will receive SelectMDx. When the SelectMDx test is positive, patients will undergo systematic biopsy. Patients with a negative SelectMDx after negative MRI will not undergo biopsy.

### Model

A decision analytical model was developed to simulate the strategies under comparison. The model starts with a decision tree, representing the diagnostic and treatment pathway of each strategy under evaluation (schematic overview in Appendix B). In the decision tree, the model stratifies the target population into groups with no cancer, Gleason 3 + 3 disease, Gleason 3 + 4 disease and Gleason ≥ 4 + 3 disease. I disease prevalence was based on studies that used systematic and targeted biopsies as the reference test (i.e., that resembles the current MRI strategy). Next, cancer detection was simulated based on the diagnostic accuracy of the included diagnostic modalities (i.e., MRI and SelectMDx). In the current MRI strategy, all patients undergo biopsy, and therefore, all cancers are detected. In the SelectMDx strategies, SelectMDx is used to select patients for MRI and/or biopsy. Therewith, the test could prevent unnecessary MRIs and biopsies and reduce detection and treatment of Gleason 3 + 3 cancers. A potential risk is that Gleason ≥ 3 + 4 are missed in SelectMDx negative cases, and consequently, treatment will be delayed.

A Markov model to simulate the consequences of the diagnostic and treatment pathway over an 18-year time horizon followed the decision tree (Appendix B). The Markov model consists of health states, and every cycle (1 year) patients can move to a different health state. Survival and long-term quality of life were included for the different health states.

### Model inputs

The model was used to synthesize various sources of evidence. Model parameters were informed by the literature estimates most relevant for a contemporary cohort of patients within the two target populations. PCa prevalence and MRI accuracy were different for both target populations. Other input parameters were assumed to be similar for the target populations. Overviews of the inputs are given in Appendix C.

#### PCa prevalence

PCa prevalence within the two target populations was based on a US study that assessed the detection rate of PCA using the current standard MRI pathway (i.e., systematic biopsies with additional targeted biopsies in case of a positive MRI) [12]. Appendix C.1 shows the PCa prevalence within two target populations.

#### MRI accuracy

The accuracy of MRI was based on the same US study that was used to estimate PCa prevalence [12]. Accuracy of MRI within the two target populations is presented in Appendix C.1

#### SelectMDx accuracy

Accuracy of SelectMDx was shown in a study that used systematic and targeted biopsies as the reference test [16]. SelectMDx accuracy for the different Gleason groups is presented in Appendix C.1

#### Treatment strategies

Distribution of treatments was based on the Comparative Effectiveness Analysis of Surgery and Radiation cohort (CEASAR) [17]. The distribution of the low risk group from CEASAR was included for the Gleason 3 + 3 cancers, while we included the distribution of treatments in the intermediate and high-risk group (weighted average) for the Gleason ≥ 3 + 4 cancers (Appendix C.1).

#### Mortality

Mortality comprised both PCa-specific mortality and other cause mortality. PCa-specific mortality was based on Gleason score and whether the disease was detected. Two scenarios were used, the SPCG-4 scenario in which PCa mortality was based on the SPCG-4 trial and the PIVOT scenario in it was based on the PIVOT trial [18, 19]. Both trials assessed PCa-specific mortality after prostatectomy and watchful waiting (i.e., no curative treatment). Weighted PCa-specific mortality after prostatectomy for intermediate-and high risk PCa was assigned to diagnosed Gleason 3 + 4 and Gleason ≥ 4 + 3 prostate cancers. Mortality for detected Gleason 3 + 3 cancers was based on the low risk cancers in the prostatectomy group. For missed PCa, the mortality of the watchful waiting groups was included. Cumulative mortality from SPCG-4 (18 years) and PIVOT (20 years) was recalculated to annual probabilities (Appendix C.2). Other cause mortality was based on annual population-based death probabilities from age 65 [20].

#### Quality of life

Quality of life was related to diagnostic strategy and treatment. To calculate quality-adjusted life years (QALYs), quality of life was expressed as a utility (valued quality of life in which 0 represents death and 1 represents perfect health). These utility values were calculated by subtracting disutilities from the maximum value of 1, as shown by Heijnsdijk et al. [21] Appendix C.3 shows the disutilities included for diagnosis and treatment. It was assumed that extra-targeted biopsies did not influence disutility for biopsy.

#### Costs

Cost assessment was performed from the health care system perspective and relevant health care costs were included. Costs were based on Medicare payments published in prior US cost-effectiveness studies. Costs were adjusted to 2021 levels, using medical cost inflation figures, when necessary [22]. Appendix C.4 provides an overview of the included costs and the literature sources.

### Analysis

For the two target populations, the consequences of all strategies with respect to the number of performed MRIs, number of performed biopsies, and number of detected prostate cancers (Gleason 3 + 3, Gleason 3 + 4, Gleason ≥ 4 + 3) were assessed. Based on these consequences, we calculated difference in QALYs and costs between the current MRI strategy and the two SelectMDx strategies. QALYs and costs were calculated using the two PCa-specific mortality scenarios (SPCG-4 and PIVOT). Appendix D shows an overview of the analyses. In sensitivity analyses, the impact of different values with respect to current detection rates, percentages of Active Surveillance in Gleason 3 + 3 cancers, PCa-specific mortality of Gleason 3 + 4 cancers and MRI accuracy was assessed.

## Results

### Population: biopsy naïve men

#### SelectMDx strategy 1: before MRI

Using SelectMDx to select patients for MRI and biopsy results in a reduction of 350 MRIs and 350 biopsies per 1000 patients compared to the current MRI strategy, while 1000 extra SelectMDx tests are performed. The detection of Gleason 3 + 3 cancers is reduced by 75 cases. These benefits come at the cost of missing 22 Gleason 3 + 4 cancers and 15 Gleason ≥ 4 + 3 cancers.

These consequences result in a gain of 0.004 QALY and a cost reduction of $1650 on average per patient under the SPCG-4 scenario over the modeled time period of 18 years (Table 1a). For the yearly population of 311,879 biopsy naïve men, this translates in an increase in QALY of about 1248 QALYs and $515 million in cost savings. In the PIVOT scenario, the QALY gain is 0.030 on average per patient with a reduction in costs of $1654, translating to 9356 QALY and $516 million for the population.

**Table 1:** QALYs and costs of the included strategies

Strategy	QALY SPCG-4	Costs SPCG-4	QALY PIVOT	Costs PIVOT
*(a) Biopsy naïve population—QALYs and costs per patient*				
Current MRI strategy	10.603	16,640	10.937	$16,760
SelectMDx 1: before MRI Difference with current strategy	+ 0.004	− $1650	+ 0.030	− $1654
SelectMDx 2:after negative MRI Difference with current strategy	+ 0.004	− $262	+ 0.006	− $263
*(b) Previous negative biopsy population—QALYs and costs per patient*				
Current MRI strategy	10.956	$10,912	11.166	$10,983
SelectMDx 1: before MRI Difference with current strategy	+ 0.006	− $1,281	+ 0.022	− $1284
SelectMDx 2:after negative MRI Difference with current strategy	+ 0.003	− $193	+ 0.004	− $194

#### SelectMDx strategy 2: after negative MRI

When SelectMDx is performed after negative MRI to select patients for systematic biopsy, 170 men undergo a SelectMDx test per 1000 men in the target population. With this strategy, the number of biopsies is reduced with 81 biopsies, and detection of Gleason 3 + 3 cancers is reduced with 12 per 1000 men in the target population. One would expect to miss one Gleason 3 + 4 cancer and one Gleason ≥ 4 + 3 cancer using this strategy.

With this strategy 0.004 QALY are gained in the SPCG-4 scenario, with $262 savings per patient (Table 1a). For the yearly population of 311,879 men, this translates to a gain of 1248 QALY and an estimated cost savings of $82 million. In the PIVOT scenario 0.006 QALY is gained and $263 are saved with SelectMDx after negative MRI, corresponding to 1871 QALYs and nearly $82 million in cost savings for the yearly population.

### Population: previous negative biopsy

#### SelectMDx strategy 1: before MRI

The number of MRIs is reduced with 434 MRIs and 434 biopsies per 1000 patients compared to the current MRI strategy. In this strategy, all 1000 patients undergo SelectMDx. The detection of Gleason 3 + 3 cancers is reduced by 50 cases. These benefits come at the cost of missing 12 Gleason 3 + 4 cancers and 11 Gleason ≥ 4 + 3 cancers.

QALY gain was 0.006 per patient with $1281 in cost savings in the SPCG-4 scenario, and a 0.022 QALY gain and $1284 cost savings in the PIVOT scenarios (Table 1b). For the yearly population with previous negative biopsies of 26,853 men, this translates into 161 QALY and 591 QALY gained in the SPCG-4 and PIVOT scenarios, respectively, with approximately $34 million in cost savings for each yearly cohort of patients.

#### SelectMDx strategy 2: after negative MRI

With SelectMDx, 182 men would receive a SelectMDx test per 1000 men. The number of biopsies is reduced by 92, and the detection of Gleason 3 + 3 cancers is reduced by 8 per 1000 men in the previous negative population. One Gleason 3 + 4 and one Gleason ≥ 4 + 3 are missed.

Using this strategy 0.003 QALY is gained in the SPCG-4 scenario with anticipated cost savings of $193 per patient. In the PIVOT scenario, the QALY gain was 0.004 with $194 in cost savings per patient (Table 1b). For the yearly population of 26,853 men, this means a QALY gain of 81 in the SPCG-4 scenario and 107 in the PIVOT scenario, with about $5 million in cost savings.

### Sensitivity analyses

Sensitivity analyses (Appendix E) showed that the percentage of patients with Gleason 3 + 3 cancers who go into active surveillance could influence the outcomes in one scenario. If active surveillance was to be used in > 60% of Gleason 3 + 3 patients, QALY is lost when SelectMDx is used before MRI in the SPCG-4 scenario. In all other scenarios, SelectMDx results in QALY gain and cost savings even when active surveillance would be used in 100% of patients with Gleason 3 + 3 cancers.

## Discussion

The results suggest that using SelectMDx for patient selection for biopsy, accounting for MRI use, improves health outcomes and saves costs in both biopsy-naïve and previous negative biopsy populations.

A previous modeling assessment showed SelectMDx's benefit in patient selection for systematic biopsy [13]. The 4 M study which compared MRI and SelectMDx showed a higher net benefit with MRI than SelectMDx alone [16]. In this study, cost-effectiveness of SelectMDx was shown when used in a combined strategy with MRI.

Several factors merit consideration. First, mortality for missed PCa was based on the watchful waiting groups (no curative treatment) from the SPCG-4 and PIVOT trials. In reality, some of the missed significant cancers would be found at curable stage. Therefore, mortality of missed disease is likely overestimated.

Second, we used a strategy in which PI-RADS 1–2 lesions received (systematic) biopsies as the ‘Current MRI strategy’ which is stated by NCCN as the most used strategy. However, some advocate for excluding systematic biopsy in PI-RADS 1–2 lesions or only perform biopsies in patients with high PSA-density. Using other strategies as the comparator will result in different outcomes since in these situations already less biopsies and treatments are performed. On the other hand, the use of SelectMDx might increase detection of significant cancers in these scenarios.

Third, we used the study of Filson et al. for the detection rates and MRI accuracy as this study also used systematic biopsies in PI-RADS 1–2 lesions. This study showed relative low detection rates, especially in the positive MRI cases and relative high numbers of cancer in PI-RADS 1–2 lesions, compared to other studies [23, 24]. In the sensitivity analyses, we showed that the overall detection rate (of 59%) could be increased to 65% (SPCG-4 scenario) and 88% (PIVOT scenario) for the SelectMDx strategy to be still dominant. Furthermore, to assess the impact of higher MRI accuracy, we performed a sensitivity analysis using data from the 4 M study [24].

Fourth, the proportion of patients with low-risk prostate cancer in active surveillance continues to increase. Nonetheless, rates of active surveillance even in contemporary series remain low. In sensitivity analyses, we demonstrated the impact of a higher percentage (i.e., 60% instead of 25%) of active surveillance. Also, we showed the threshold of active surveillance at which SelectMDx still resulted in both health gain and cost savings. With 60% active surveillance, QALYs are lost under the SPCG-4 scenario when SelectMDx would be used before MRI. In all other strategies and scenarios, SelectMDx resulted in health gain and cost savings, even when active surveillance was used in all patients with Gleason 3 + 3 cancers.

Fifth, we used the same diagnostic accuracy of SelectMDx for patients with PI-RADS 1–2 as for the total group. In practice, this accuracy could be different resulting in different outcomes.

Last, this assessment is performed in the context of the US with input data that was aimed to provide a general picture for the US. As input data for the model will differ in other countries, the results of this assessment are only to a limited extent generalizable to other countries. Furthermore, the results will differ as well when the cost-effectiveness of SelectMDx will be assessed in the context of a specific US center.

## Conclusion

This study shows that SelectMDx could have value in reducing overdiagnosis and overtreatment without excessively compromising the detection of significant cancers when used in MRI strategies. Using SelectMDx before MRI to select patients for MRI and biopsy resulted in higher cost savings and higher impact on health outcomes compared to using SelectMDx after negative MRI.

## Supplementary Information

Below is the link to the electronic supplementary material.

Supplementary file1 (DOCX 1158 KB)

## Data Availability

All input data used for this modelling study is included in the published article (and supplementary files).
