# Supplementary material for: Cost-effectiveness of an urinary biomarker panel in combination with MRI for prostate cancer diagnosis
Source: World J Urol. 2023 May 3;41(6):1527–32. doi: 10.1007/s00345-023-04389-w (PMC10241729; doi:10.1007/s00345-023-04389-w)
Supplement: Supplementary file 1 — Supplementary file1 (DOCX 1158 KB) [file 345_2023_4389_MOESM1_ESM.docx]

**Appendix A: Strategies**


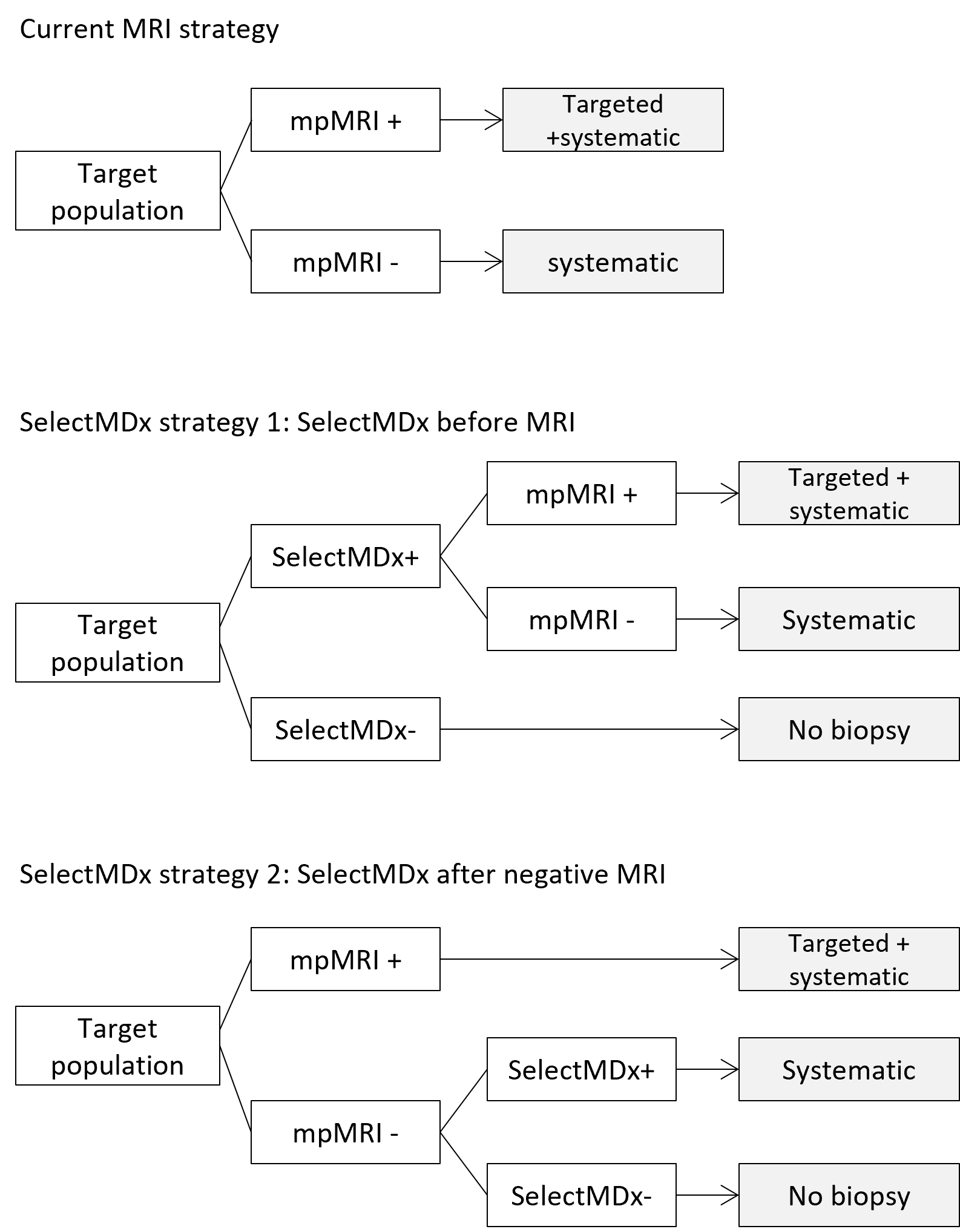


**Appendix B: structure of the model**

B.1 Decision tree current care


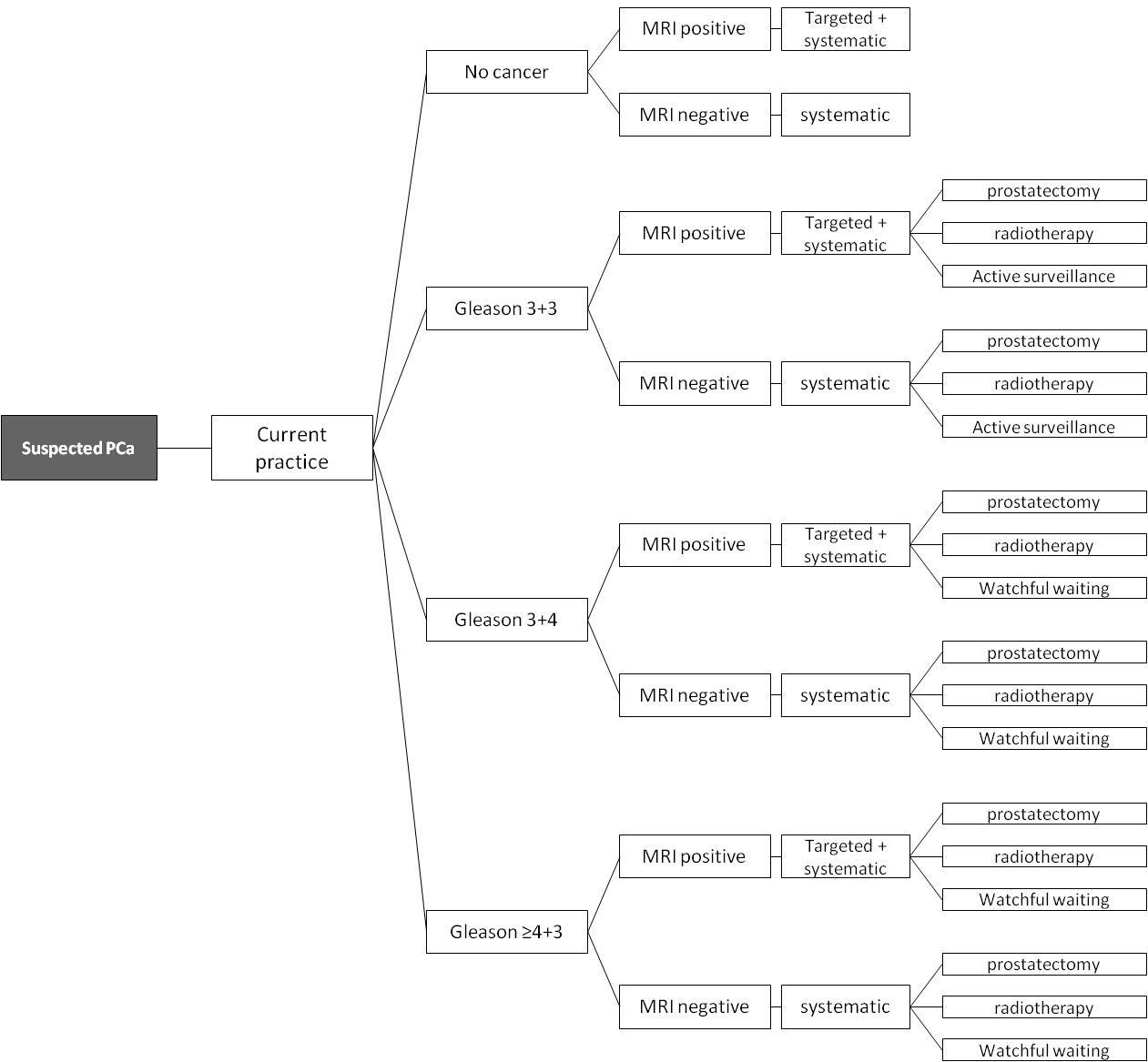


B.2 Decision tree SelectMDx strategy 1: before MRI


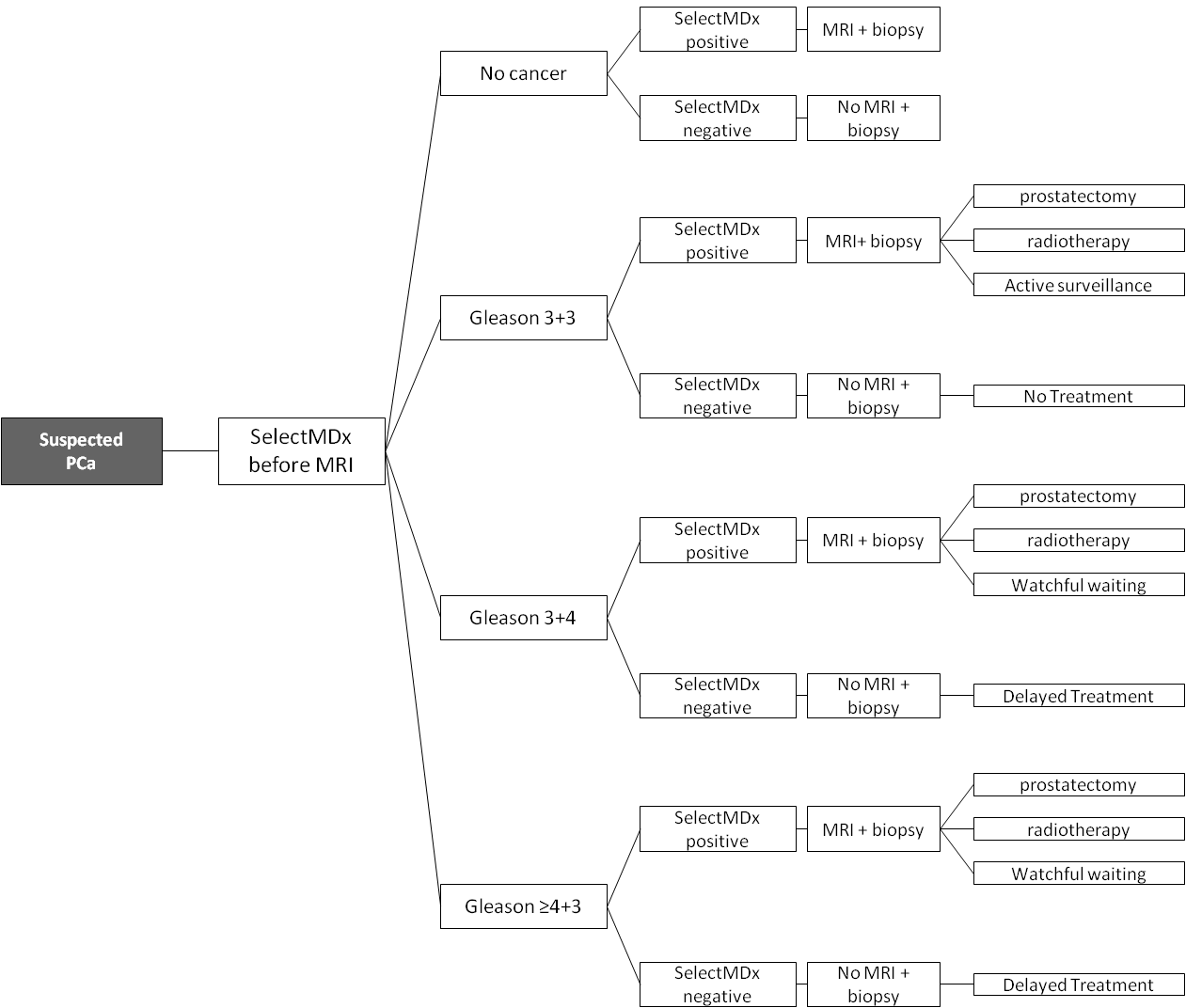


B.3 Decision tree SelectMDx strategy 2: after negative MRI


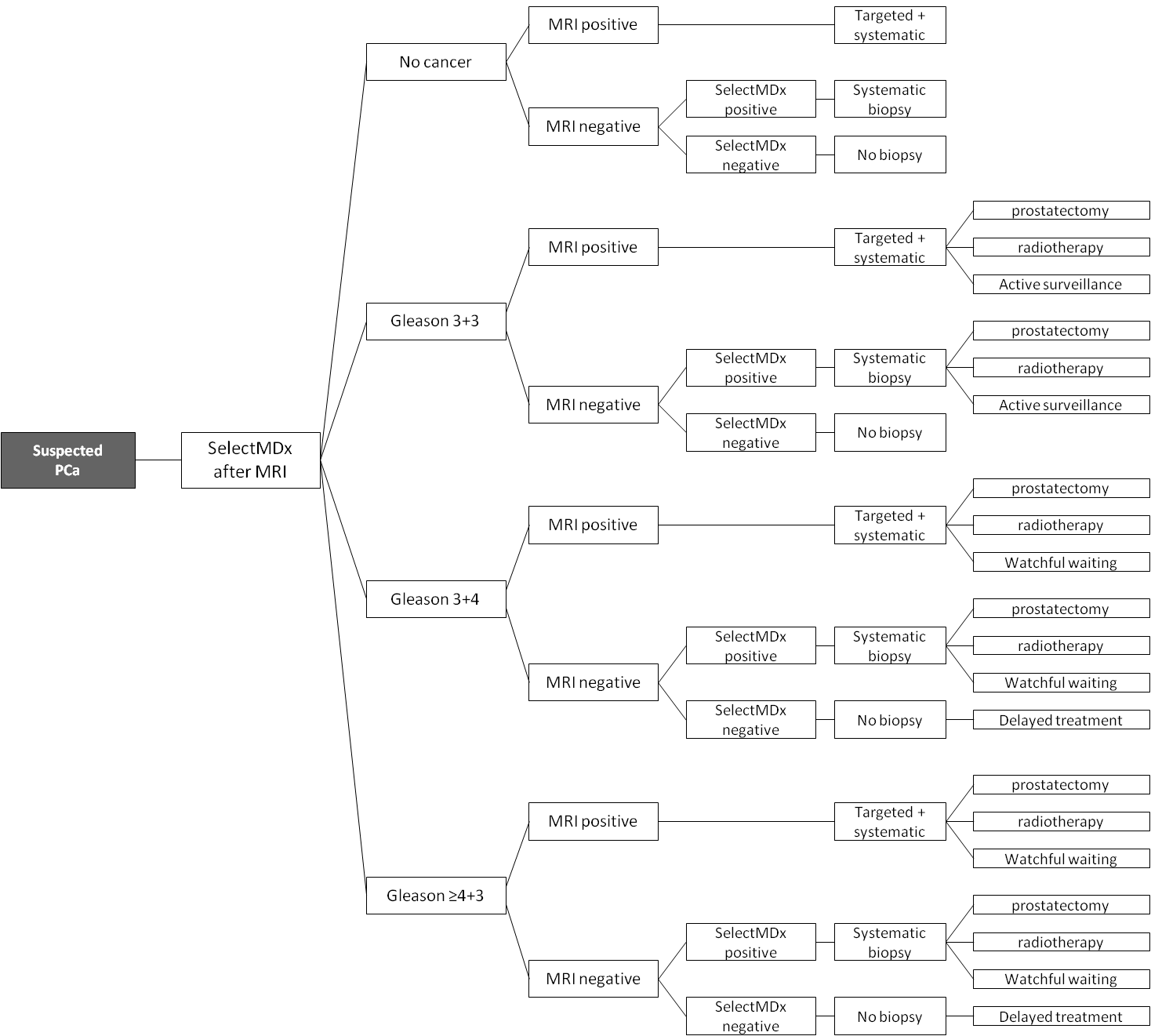


B.4: Markov part of the model: The same Markov structure was used for all three strategies.


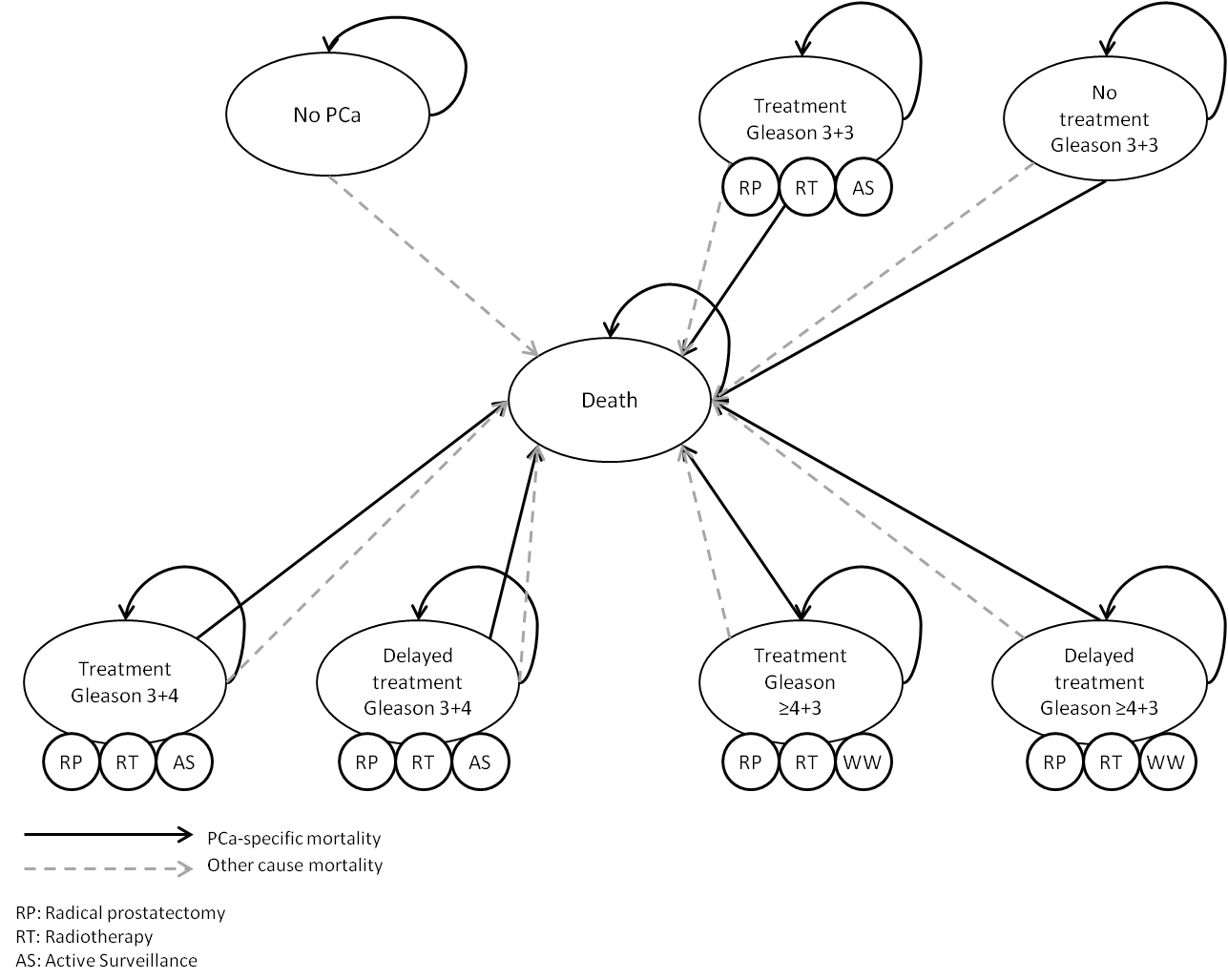


**Appendix C: Input**

C1: Transition probabilities

| Parameter | Biopsy naïve population | Previous negative population | Source |
| --- | --- | --- | --- |
| Percentage no cancer | 41.0% | 62.3% | Filson et al.[1] |
| Percentage Gleason 3+3 | 21.6% | 14.5% | Filson et al.[1] |
| Percentage Gleason 3+4 | 19.8% | 10.5% | Filson et al.[1] |
| Percentage Gleason ≥4+3 | 17.6% | 12.7% | Filson et al.[1] |
|  |  |  |  |
| MRI positive in case of no PCa | 71.9% | 76.7% | Filson et al.[1] |
| MRI positive in case of Gleason 3+3 | 84.5% | 85.1% | Filson et al.[1] |
| MRI positive in case of Gleason 3+4 | 92.1% | 91.2% | Filson et al.[1] |
| MRI positive in case of Gleason ≥4+3 | 94.8% | 95.1% | Filson et al.[1] |
|  |  |  |  |
| SelectMDx positive in case of no PCa | 42.1% | | Hendriks et al.[2] |
| SelectMDx positive in case of Gleason 3+3 | 65.2% | | Hendriks et al.[2] |
| SelectMDx positive in case of Gleason 3+4 | 88.8% | | Hendriks et al.[2] |
| SelectMDx positive in case of Gleason ≥4+3 | 91.5% | | Hendriks et al.[2] |
|  |  |  |  |
| *Treatment Gleason 3+3* |  |  |  |
| Radical prostatectomy | 48.2% | | Eifler et al.[3] |
| Radiotherapy | 26.4% | | Eifler et al.[3] |
| Active Surveillance | 25.4% | | Eifler et al.[3] |
|  |  |  |  |
| *Treatment Gleason* ≥3+4 |  |  |  |
| Radical prostatectomy | 54.3% | | Eifler et al.[3] |
| Radiotherapy | 40.2% | | Eifler et al.[3] |
| Watchful waiting | 5.5% | | Eifler et al.[3] |

C.2 Mortality

| Parameter | SPCG-4 scenario –  annual PCa-specific mortality | PIVOT scenario –  annual PCa-specific mortality |
| --- | --- | --- |
| Detected Gleason 3+3 | 0.6% | 0.2% |
| Missed Gleason 3+3 | 0.8% | 0.3% |
| Detected Gleason 3+4* | 1.4% | 1.0% |
| Missed Gleason 3+4* | 2.6% | 0.5% |
| Detected Gleason ≥4+3 | 1.4% | 1.0% |
| Missed Gleason ≥4+3 | 2.6% | 0.% |

C.3 Utility values

| Parameter | Disutility | Calculation (when applicable) |
| --- | --- | --- |
| Biopsy | 0.006 | (3/52)*0.1 |
| Cancer diagsnosis | 0.017 | (1/12)*0.2 |
| Radiotherapy (first year) | 0.228 | (2/12)*0.27+(10/12*0.22) |
| Radical prostatectomy (first year) | 0.247 | (2/12)*0.33+(10/12*0.23) |
| Active surveillance | 0.03 |  |
| Post recovery period  (RT and RP) | 0.05 |  |

| Parameter | Value ($) | Calculation | Source |
| --- | --- | --- | --- |
| Biopsy | 1481 | Average of costs presented in 7 studies | 7 studies[4-10] |
| Prostate MRI | 826 | Average of costs presented in 4 studies | 4 studies[5, 7-9] |
| SelectMDx test | 785 |  | MDxHealth |
| Radical Prostatectomy | 14,630 |  | Wang et al.[4] |
| Radiotherapy | 29,334 |  | Wang et al.[4] |
| Total AS/WW per year | 564 |  | Roth et al.[6] |
| Biopsy complications | 498 | 3%*$16,610 | Gershman et al.[11], Hayes et al.[12] |
| RP complications first year  -Surgical complications  -Urine incontinence  -Erectile dysfunction  Total RP first year | 19182^1^  208  1047  3,171 | 16%^2^*$1,299  54%^2^*$1,938 | Cooperberg et al.[13]  Cooperberg et al.[13]  Cooperberg et al.[13] |
| RP complications after first year  -Urine incontinence -Erectile dysfunction  Total RP after first year | 72  215  287 | 9.3%^3^*$776  31%^3^*$694 | Cooperberg et al.[13]  Cooperberg et al.[13] |
| RT complications first year  -Acute toxicity -Gastrointestinal toxicity -Genitourinary toxicity -Erectile dysfunction Total RT first year | 488  30 55 627 1,201 | 2.1%*$1,409 2.9%*$1,905 32.4%^4^*$1,938 | Cooperberg et al.[13]  Cooperberg et al.[13] Cooperberg et al.[13]  Cooperberg et al.[13] |
| RT complications after first year -Gastrointestinal toxicity -Genitourinary toxicity -Erectile dysfunction Total RT after first year | 230 55 293 378 | 2.1%*$1,409 2.9%*$1,905 42.2%^5^*$694 | Cooperberg et al.[13]  Cooperberg et al.[13]  Cooperberg et al.[13] |

C.4 Costs

^1^Weighted average of 85% robotic and 15% open surgery
^2^Probability at 6 months, weighed average of 85% robotic and 15% open surgery
^3^Probability at 24 months, weighed average of 85% robotic and 15% open surgery
^4^Probability at 6 months, weighed average of 70% IMRT, 18% Brachytherapy and 12% EBRT (3DCRT)
^5^Probability at 24 months, weighed average of 70% IMRT, 18% Brachytherapy and 12% EBRT (3DCRT)

Distibutions of treatments (robot/open and IMRT/Brachytherapy/EBRT) are based on expert opinion

**Appendix D: Analyses overview**

**
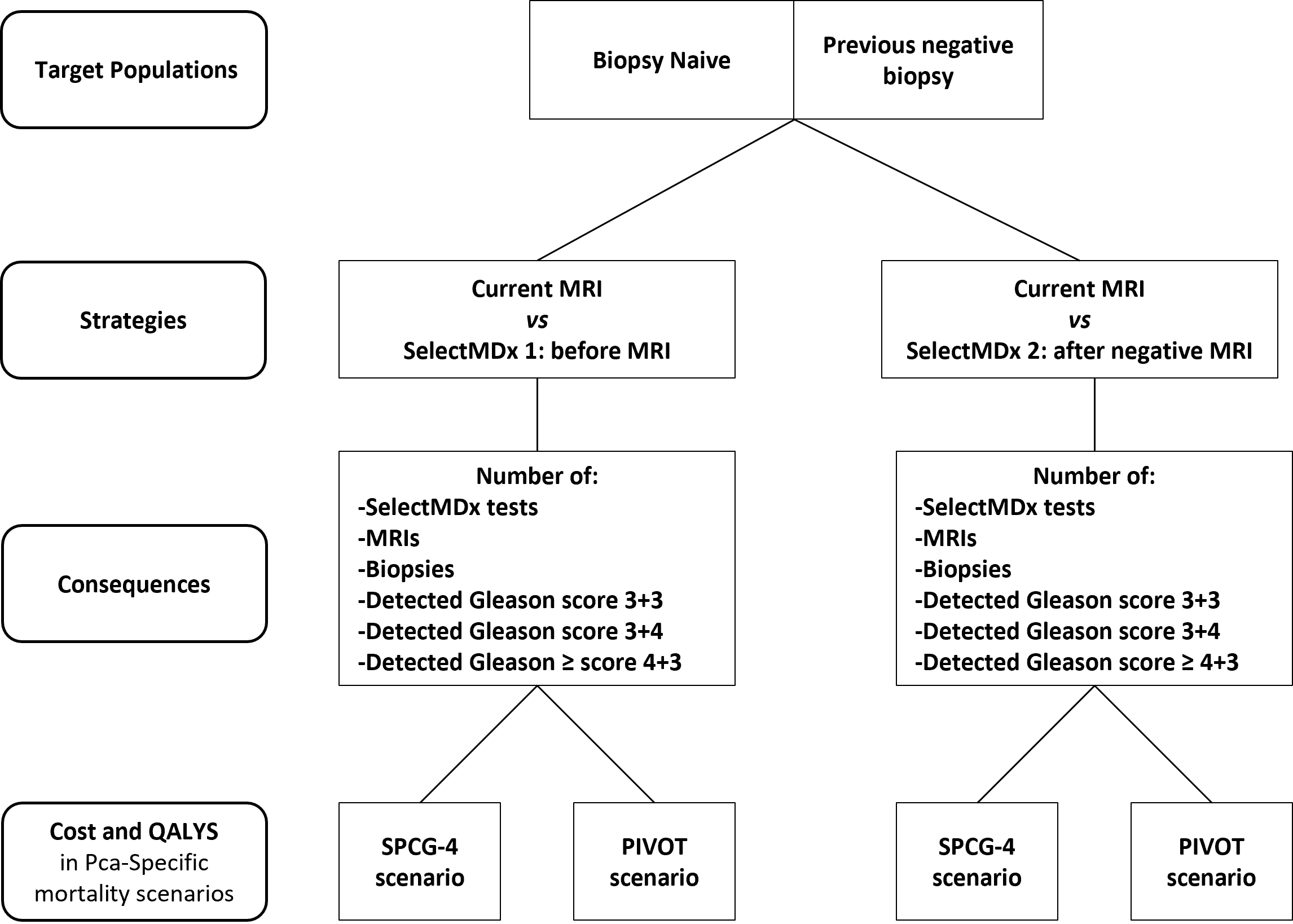
**

**Appendix E: sensitivity analyses**

For both the biopsy naïve population and the population with a previous negative biopsy we assessed the impact of different values for input parameters in both scenarios (SPCG-4 and PIVOT). We performed these analyses on the comparison between the two SelectMDx strategies with the current MRI strategy. The following sensitivity analyses were performed:

**-Treshold analysis of the percentage of patients in the population that has prostate cancer and the percentage of these cancers that is significant.** This treshold analysis was performed since the percentage of patients with prostate cancer, and the percentage of these cancers that is significant, can differ between centers. This treshold analysis shows the maximum percentage of patients with prostate cancer, and the maximum percentage of these cancers that is significant for which SelectMDx is dominant (i.e. results in health gain and cost savings). For this sensitivity analysis we assumed the same value for the percentage of cancers and the percentage that is significant (e.g. 60% PCa of which 60% is significant). Significant PCa was defined as Gleason ≥3+4.

-**Sensitivity analysis with a higher percentage of patients with Gleason 3+3 cancers that go into active surveillance**. This sensitivity analysis was performed since the percentage of patients in AS is increasing in the last years, while the study from which the percentage of patients in AS was obtained, collected their data between 2011 and 2012. This analysis was performed using the highest estimate of the percentage of AS in Gleason 3+3 cancers that was given by urologists in the US.

**-Treshold analysis of the percentage of patients with Gleason 3+3 cancers that go into active surveillance.** Higher percentages of patients with Gleason 3+3 cancers in active surveillance (and therewith lower overtreatment) result in lower QALY and costs gains with SelectMDx. This threshold analysis shows the maximum percentage of patients in active surveillance for which SelectMDx is still dominant (i.e. results in health gain and cost savings).

**-Sensitivity analysis with a lower PCa specific mortality for Gleason 3+4 cancers.** This sensitivity analysis was performed since it is known that Gleason 3+4 cancers have a lower mortality compared to Gleason ≥4+3, while in the base analysis the same mortality was included for these cancers. This analysis was performed by including the PCa-specific mortality of low risk cancers (From PIVOT and SPCG-4 data) for Gleason 3+4 cancers.

-**Sensitivity analyses with other MRI accuracy**. This sensitivity analyses was performed since MRI accuracy differs between centers and this impacts the cost-effectiveness. In this sensitivity analyses we used the accuracy of MRI from the Dutch ‘4M study’ which shows relative high MRI accuracy.[14] This other MRI accuracy only impacts the results in the SelectMDx after MRI scenario.

Biopsy naïve population

*SelectMDx strategy 1: Before MRI*

| Analysis | Outcome | SPCG-4 scenario | PIVOT scenario |
| --- | --- | --- | --- |
| Base case analysis | QALY difference with current strategy | +0.004 | +0.030 |
|  | Cost difference with current strategy | -$1,650 | -$1,654 |
| Treshold analysis: PCa Prevalence | Maximum percentage of PCa and Significant PCa for Which SelectMDx is dominant | 65%/65% | 83%/83% |
| AS in Gleason 3+3: 60% | QALY difference with current strategy | -0.007 | +0.019 |
|  | Cost difference with current strategy | -$1,151 | -$1,157 |
| Treshold analysis:  AS in Gleason 3+3 | Maximum Percentage of AS for which SelectMDx is dominant | 33% | 100% |
| Low risk mortality for Gleason 3+4 | QALY difference with current strategy | +0.022 | +0.038 |
|  | Cost difference with current strategy | -$1,644 | -$1,661 |

*SelectMDx strategy 2: After negative MRI*

| Analysis | Outcome | SPCG-4 scenario | PIVOT scenario |
| --- | --- | --- | --- |
| Base case analysis | QALY difference with current strategy | +0.004 | +0.006 |
|  | Cost difference with current strategy | -$262 | -$263 |
| Treshold analysis: PCa Prevalence | Maximum percentage of PCa and Significant PCa for Which SelectMDx is dominant | 87%/87% | 95%/95% |
| AS in Gleason 3+3: 60% | QALY difference with current strategy | +0.002 | +0.005 |
|  | Cost difference with current strategy | -$185 | -$187 |
| Treshold analysis:  AS in Gleason 3+3 | Maximum Percentage of AS for which SelectMDx is dominant | 100% | 100% |
| Low risk mortality for Gleason 3+4 | QALY difference with current strategy | +0.005 | +0.007 |
|  | Cost difference with current strategy | -$262 | -$263 |
| MRI accuracy data from 4M study | QALY difference with current strategy | +0.015 | +0.019 |
|  | Cost difference with current strategy | -$771 | -$775 |

Previous negative biopsy population

*SelectMDx strategy 1: Before MRI*

| Analysis | Outcome | SPCG-4 scenario | PIVOT scenario |
| --- | --- | --- | --- |
| Base case analysis | QALY difference with current strategy | +0.006 | +0.022 |
|  | Cost difference with current strategy | -$1,281 | -$1,284 |
| Treshold analysis: PCa Prevalence | Maximum percentage of PCa and Significant PCa for Which SelectMDx is dominant | 66%/66% | 83%/83% |
| AS in Gleason 3+3: 60% | QALY difference with current strategy | -0.001 | +0.015 |
|  | Cost difference with current strategy | -$1,007 | -$1,011 |
| Treshold analysis:  AS in Gleason 3+3 | Maximum Percentage of AS for which SelectMDx is dominant | 53% | 100% |
| Low risk mortality for Gleason 3+4 | QALY difference with current strategy | +0.016 | +0.026 |
|  | Cost difference with current strategy | -$1,277 | -$1,288 |

*SelectMDx strategy 2: After negative MRI*

| Analysis | Outcome | SPCG-4 scenario | PIVOT scenario |
| --- | --- | --- | --- |
| Base case analysis | QALY difference with current strategy | +0.003 | +0.004 |
|  | Cost difference with current strategy | -$193 | -$194 |
| Treshold analysis: PCa Prevalence | Maximum percentage of PCa and Significant PCa for Which SelectMDx is dominant | 80%/80% | 91%/91% |
| AS in Gleason 3+3: 60% | QALY difference with current strategy | +0.002 | +0.003 |
|  | Cost difference with current strategy | -$140 | -$141 |
| Treshold analysis:  AS in Gleason 3+3 | Maximum Percentage of AS for which SelectMDx is dominant | 100% | 100% |
| Low risk mortality for Gleason 3+4 | QALY difference with current strategy | +0.004 | +0.005 |
|  | Cost difference with current strategy | -$190 | -$191 |
| MRI accuracy data from 4M study | QALY difference with current strategy | +0.011 | +0.014 |
|  | Cost difference with current strategy | -$609 | -$613 |

**Appendix - References**

1. Filson CP, Natarajan S, Margolis DJ, Huang J, Lieu P, Dorey FJ, et al. Prostate cancer detection with magnetic resonance-ultrasound fusion biopsy: The role of systematic and targeted biopsies. Cancer. 2016;122(6):884-92.

2. Hendriks RJ, van der Leest MMG, Israel B, Hannink G, YantiSetiasti A, Cornel EB, et al. Clinical use of the SelectMDx urinary-biomarker test with or without mpMRI in prostate cancer diagnosis: a prospective, multicenter study in biopsy-naive men. Prostate cancer and prostatic diseases. 2021;24(4):1110-9.

3. Eifler JB, Alvarez J, Koyama T, Conwill RM, Ritch CR, Hoffman KE, et al. More Judicious Use of Expectant Management for Localized Prostate Cancer during the Last 2 Decades. The Journal of urology. 2017;197(3 Pt 1):614-20.

4. Wang SY, Wang R, Yu JB, Ma X, Xu X, Kim SP, et al. Understanding regional variation in Medicare expenditures for initial episodes of prostate cancer care. Medical care. 2014;52(8):680-7.

5. Altok M, Kim B, Patel BB, Shih YT, Ward JF, McRae SE, et al. Cost and efficacy comparison of five prostate biopsy modalities: a platform for integrating cost into novel-platform comparative research. Prostate cancer and prostatic diseases. 2018.

6. Roth JA, Gulati R, Gore JL, Cooperberg MR, Etzioni R. Economic Analysis of Prostate-Specific Antigen Screening and Selective Treatment Strategies. JAMA oncology. 2016;2(7):890-8.

7. Barnett CL, Davenport MS, Montgomery JS, Wei JT, Montie JE, Denton BT. Cost-effectiveness of magnetic resonance imaging and targeted fusion biopsy for early detection of prostate cancer. BJU international. 2018;122(1):50-8.

8. Pahwa S, Schiltz NK, Ponsky LE, Lu Z, Griswold MA, Gulani V. Cost-effectiveness of MR Imaging-guided Strategies for Detection of Prostate Cancer in Biopsy-Naive Men. Radiology. 2017:162181.

9. Lotan Y, Haddad AQ, Costa DN, Pedrosa I, Rofsky NM, Roehrborn CG. Decision analysis model comparing cost of multiparametric magnetic resonance imaging vs. repeat biopsy for detection of prostate cancer in men with prior negative findings on biopsy. Urol Oncol. 2015;33(6):266 e9-16.

10. Sathianathen N, Konety B, Alarid-Escudero F, Lawrentschuk N, Kuntz K. Active Surveillance Follow-up Strategies: A Cost-Effectiveness Analysis. J Urology. 2018;199(4):E209-E.

11. Gershman B, Van Houten HK, Herrin J, Moreira DM, Kim SP, Shah ND, et al. Impact of Prostate-specific Antigen (PSA) Screening Trials and Revised PSA Screening Guidelines on Rates of Prostate Biopsy and Postbiopsy Complications. European urology. 2016.

12. Hayes JH, Ollendorf DA, Pearson SD, Barry MJ, Kantoff PW, Lee PA, et al. Observation versus initial treatment for men with localized, low-risk prostate cancer: a cost-effectiveness analysis. Annals of internal medicine. 2013;158(12):853-60.

13. Cooperberg MR, Ramakrishna NR, Duff SB, Hughes KE, Sadownik S, Smith JA, et al. Primary treatments for clinically localised prostate cancer: a comprehensive lifetime cost-utility analysis. BJU international. 2013;111(3):437-50.

14. van der Leest M, Cornel E, Israel B, Hendriks R, Padhani AR, Hoogenboom M, et al. Head-to-head Comparison of Transrectal Ultrasound-guided Prostate Biopsy Versus Multiparametric Prostate Resonance Imaging with Subsequent Magnetic Resonance-guided Biopsy in Biopsy-naive Men with Elevated Prostate-specific Antigen: A Large Prospective Multicenter Clinical Study. European urology. 2019;75(4):570-8.
